# Shell biomechanics suggests an aquatic palaeoecology at the dawn of turtle evolution

**DOI:** 10.1038/s41598-024-72540-7

**Published:** 2024-09-18

**Authors:** Gabriel S. Ferreira, Guilherme Hermanson, Christina Kyriakouli, Dawid Dróżdż, Tomasz Szczygielski

**Affiliations:** 1 Senckenberg Centre for Human Evolution and Palaeoenvironment, University of Tübingen, Hölderlinstraße 12, 72074 Tübingen, Germany; 2 Fachbereich Geowissenschaften, Eberhard Karls Universität Tübingen, Hölderlinstraße 12, 72074 Tübingen, Germany; 3 Department of Geosciences, University of Fribourg, Chemin du Musée 6, 1700 Fribourg, Switzerland; 4 Nalecz Institute of Biocybernetics and Biomedical Engineering, Polish Academy of Sciences PL, Ks. Trojdena 4, 02-109 Warsaw, Poland; 5 Institute of Paleobiology, Polish Academy of Sciences PL, Twarda 51/55, 00-818 Warsaw, Poland

**Keywords:** Palaeobiology, Finite element analyses, Geometric morphometrics, Testudinata, Testudines, Triassic, Palaeontology, Biomechanics

## Abstract

The turtle shell is a remarkable structure that has intrigued not only evolutionary biologists but also engineering and material scientists because of its multi-scale complexity and various functions. Although protection is its most apparent role, the carapace and plastron are also related to many physiological functions and their shape influences hydrodynamics and self-righting ability. As such, analysing the functional morphology of the shell could help understanding the ecology of Triassic stem-turtles, which will contribute to the century-long debate on the evolutionary origins of turtles. Here, we used 3D imaging techniques to digitize the shells of two of the earliest stem-turtle taxa, *Proganochelys* and *Proterochersis*, and submitted their models to biomechanical and shape analyses. We analysed the strength performance under five predation scenarios and tested the function of two morphological traits found in stem-turtles, the epiplastral processes and an attached pelvic girdle. The latter, also present in the crown-lineage of side-necked turtles, has been suggested to increase load-bearing capacity of the shell or to improve swimming in pleurodires. Our results do not confirm the shell-strengthening hypothesis and, together with the results of our shape analyses, suggest that at least one of the first stem-turtles (*Proterochersis*) was an aquatic animal.

## Introduction

The turtle shell is a complex structure of great interest to evolutionary biologists^1,2^ and engineering scientists^3–5^ alike. Multi-scale complexity is observed in the nanostructure—in the nature and arrangement of its molecular constituents—and the microstructure—internal bone and connective tissue architecture. Also in the macrostructure^6^, although the layering of compact and trabecular bone of the shell bones is also seen in other armoured animals (e.g., in the osteoderms of armadillos and crocodiles^7,8^), the serial arrangement of endochondral bone elements (the neurals and costals) scaffolded by dermal bone plates (the peripherals, nuchal, suprapygal, and pygal)^9^, in contact via interdigitated sutures joined by collagenous fibres^6^ is unique to the turtle shell. Even though protection is its most evident function, the properties of the turtle shell also affect the animal’s buoyancy and hydrodynamics^10^ and serve as an ionic and metabolic reservoir^3^. The morphological complexity and functional diversity have contributed to the continuing interest in the evolutionary origins of the turtle shell^1,11–13^.

**Fig. 1:**
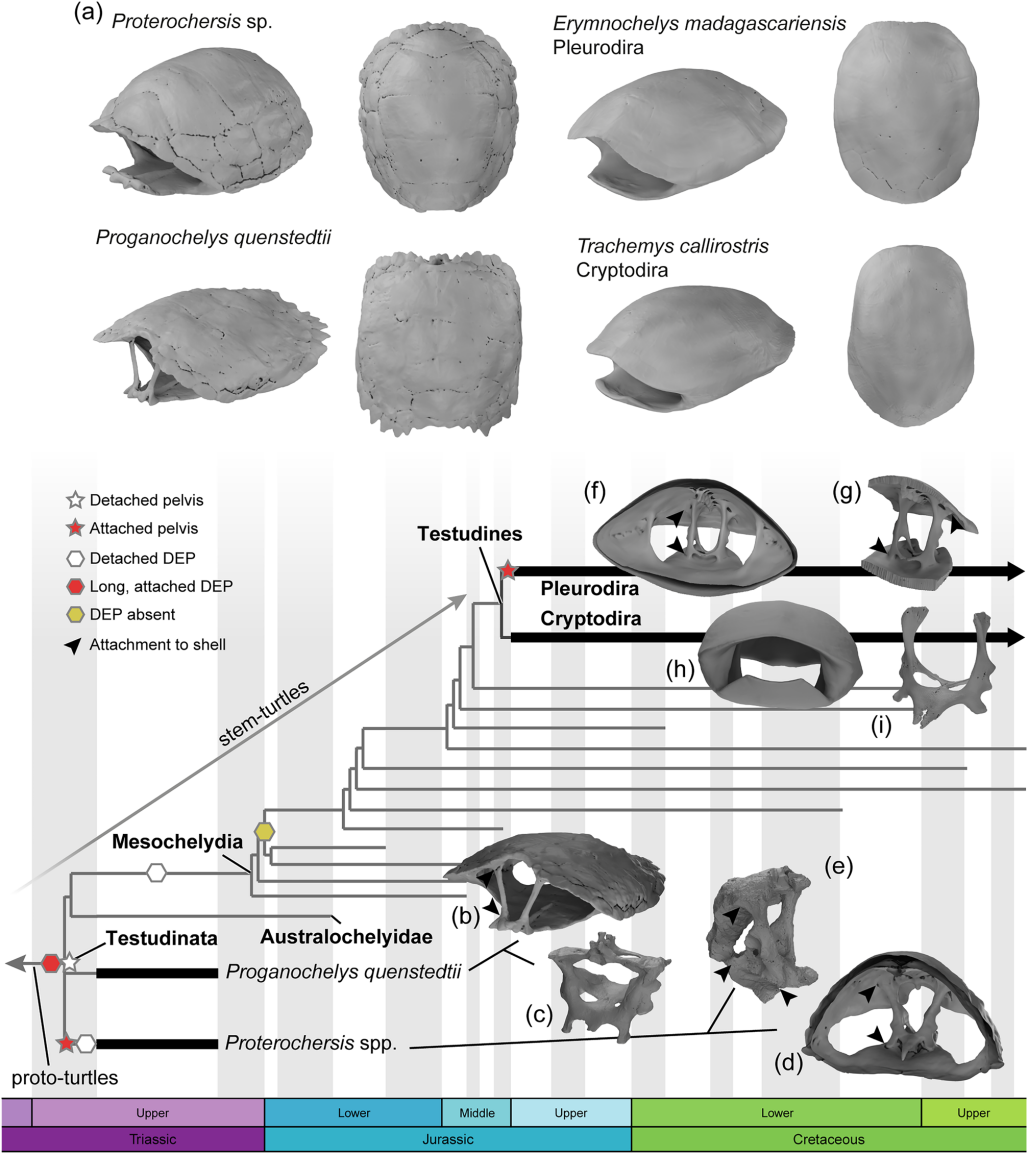
Summary of the relationship of taxa analysed here and their relevant morphological traits. (**a**) 3D renderings of their shells in anterolateral (left) and dorsal (right) views. (**b**, **c**) *Proganochelys quenstedtii* models showing the epiplastral processes (**b**) and pelvic girdle (**c**). Shells (**d**, **f**, **h**) and isolated pelvic girdles (**e**, **g**, **i**) of *Proterochersis* (only posterior shell; (**d**, **e**), *Erymnochelys madagascariensis* (only posterior shell; (**f**, **g**), and *Trachemys callirostris* (**h**, **i**). Black arrows show attachments of the dorsal epiplastral processes (DEP) (**b**) and pelvic girdles (**e**–**g**) to the shell. Light and dark grey branches in the tree represent the stem-lineage and the crown-group Testudines, respectively.

Despite a “complete turtle shell” being the defining character of Testudinata^14^, early (Triassic) stem-turtles (Fig. 1) had many distinct traits in comparison to later mesochelydians and to their extant relatives (Testudines)^14^. For example, ankylosed (fused) shell bones, robust connections with limb girdles and associated structures, a larger number of costal, neural, and peripheral bones, and, in some species, a mosaic of supernumerary osteoderms, are common in stem-turtles^11,15–19^. Large, rod-like dorsal processes of the epiplastra, likely homologous to the clavicles of other reptiles^16,20^ and inherited from their non-shelled ancestors^21–23^ also occur in early testudinates (Fig. 1). In the Proterochersidae, these processes were long but did not reach the carapace^24–26^. However, in the Australochelyidae and *Proganochelys quenstedtii*, they formed wide sutural contacts in the nuchal region (Fig. 1b), on each side of the neck^16,17^. Those epiplastral processes were significantly reduced and eventually lost in post-Triassic taxa. The connection between the pelvic girdle and the shell is also variable in stem-turtles (Table 1; Fig. 1). In adult proterochersids it is completely fused to the plastron and carapace (Fig. 1d-e)^15,24–26^. The pelvic morphology is very similar in australochelyids, but despite earlier reports^17,27^, there was likely no sutural attachment to the shell^13,28^, although a tight articulation is observed^17,27^. In *Proganochelys quenstedtii*, the pelvis is separate from the shell (Fig. 1c), and the carapace forms platform-like processes receiving the dorsal extremities of the ilia^16,29^.

**Table 1:** Relationships between limb girdles and shell in Triassic and extant turtles.

	Epiplastral process	Ilium	Pubis	Ischium
Proterochersidae	Free	Fused to carapace along entire length	Fused to plastron	Fused to plastron
*Proganochelys quenstedtii*	Sutured to carapace	Articulated dorsally with a descending platform of carapace	Loosely articulated with plastron	Posterior to plastron
Australochelyidae	Sutured to carapace	Tightly articulated with carapace, posterior end free	Tightly articulated with plastron	Tightly articulated with plastron
Cryptodira	Absent	Loosely articulated with carapace	Loosely articulated with plastron	Loosely articulated with plastron, may be posterior to plastron
Pleurodira	Absent	Sutured to carapace along entire length	Sutured to plastron, epipubic process and pubic plate reduced	Sutured to plastron, ischiadic plate reduced

The epiplastral processes are unique to stem-turtles, but pelvic girdle anatomy is one of the most noticeable skeletal features distinguishing the two major lineages of Testudines. In side-necked turtles (Pleurodira) the pelvis forms sutural attachments with the shell (Fig. 1f–g) whereas in hidden-necked turtles (Cryptodira) it is merely articulated to the shell (Fig. 1h–i)^16,30–32^. This ligamentous articulation of the pelvis in cryptodires enables a range of movement similar to those of other vertebrates^33^. Conversely, the rigid attachment of the pelvic girdles in pleurodires renders them motionless during locomotion, limiting femoral protraction^33,34^. Although beneficial to terrestrial locomotion, increased hindlimb protraction has been shown to reduce stability during swimming^33^ and thus the attached pelvic girdle could be an adaptation to more aquatic lifestyles (hereafter we use aquatic to refer to both fully and semiaquatic taxa, in other words, non-terrestrial turtles). Indeed, pleurodires are more stable and show greater turning performance than cryptodires in aquatic media^35^. The attached pelvic girdle has been also shown to participate in stress distribution in some loading scenarios^36^, also potentially related to increased shell strength and flatter phenotypes in pleurodires^37^. The sutural attachment of the pelvis is already partially (*Platychelys oberndorferi*^38^) or fully (*Notoemys* spp.^39,40^) present in the earliest known (Late Jurassic) aquatic representatives of the pleurodiran lineage.

Aside from its physiological functions, the shell shape is also subjected to different selective pressures in extant turtles with distinct lifestyles. The functional performance of the shell in the ability to self-right (more associated with terrestrial turtles^41^), hydrodynamic efficiency and strength together have been shown to predict the ecology of living species with great accuracy^42–44^. Hence, understanding the functioning of the shells of early stem-turtles might shed some light on their palaeobiology and early evolution. Moreover, the role of the variable osseous attachments between the girdles and the shell in Triassic stem-turtles was never established. Among possible explanations, three hypotheses about their function can be formulated: (i) stabilisation of the locomotor apparatus after the severe reorganisations caused by the development of the shell^1^ and before the girdles and limbs attained their derived morphology; (ii) aiding limb function in particular environments (terrestrial vs. aquatic) and/or locomotor scenarios (e.g., swimming vs. bottom walking) and allowing the occupation of new ecological niches; and (iii) serving as mechanical support within the shell, improving the defence against large predators diversifying during the Late Triassic. Here, we conduct Finite Element Analysis (FEA) to test whether the epiplastral processes and pelvic girdles are related to increasing shell strength (hypothesis iii). FEA has been extensively employed in both biology^36,43^ and palaeontology^45–47^, because this method allows simulating specific loading scenarios to test biomechanical hypotheses. We also used geometric morphometrics to further explore the implications of overall shell shape to the palaeobiology of two of the earliest stem-turtles, *Proganochelys* and *Proterochersis*. Our results help to understand the function of the epiplastral processes and attached pelvic girdles in Triassic testudinates and provide further evidence for an aquatic lifestyle in proterochersids, contributing to our understanding of the origin of the turtle shell.

## Results

### Biomechanical simulations

**Fig. 2:**
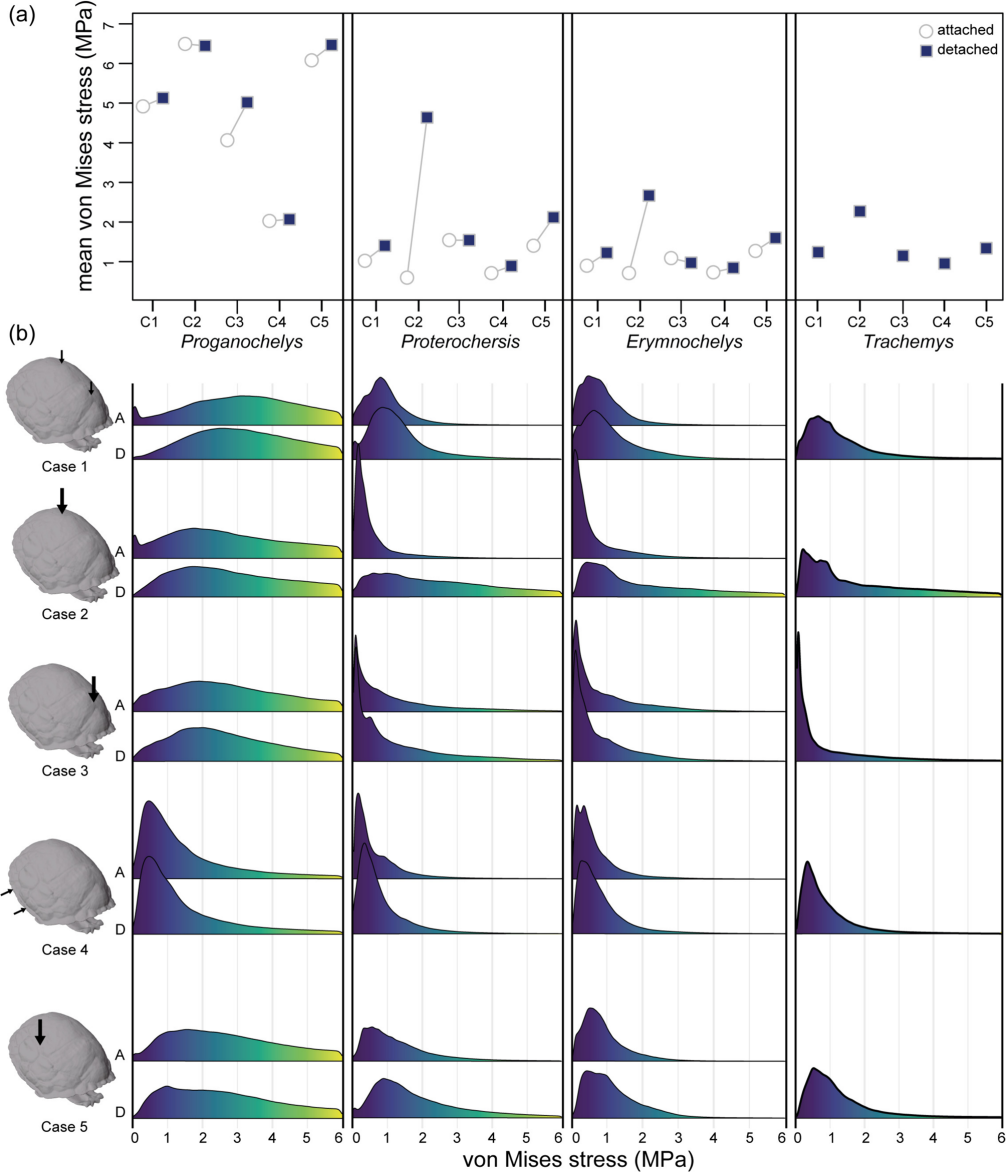
(**a**) Mean stress per element and (**b**) stress magnitude distributions (from left to right columns) *Proganochelys quenstedtii*, *Proterochersis* sp., the pleurodire *Erymnochelys madagascariensis* and the cryptodire *Trachemys callisrostris*. Different cases are represented by labels C1 to C5 in (**a**) and by different rows in (**b**). Results from simulations on the attached and detached models are respectively identified by white circles and dark blue squares in (**a**) and by S and D in (**b**).

To test whether attaching the epiplastral processes or pelvic girdles is related to increasing shell strength, we ran two sets of finite element analyses in models of four turtle taxa (Fig. 1a): *Proganochelys*, *Proterochersis*, the pleurodire *Erymnochelys madagascariensis*, and the cryptodire *Trachemys callirostris*. In the “attached” set we analysed models with the original morphology and in the “detached” models we removed the connection of the structure of interest (the epiplastral processes in *Proganochelys* and the pelvic girdles in *Proterochersis* and *E. madagascariensis*) and then applied loads at different positions, to simulate five predation scenarios (see below). The simulation design allows us to assess if attaching such parts results in a less stressed (and hence stronger) shell. The analyses show no clear sign of stress reduction related to suturing the epiplastral processes or pelvic girdle to the shell. Mean stress per element (Fig. 2a) decreases only slightly in the attached models when compared to the detached models in cases 1 (midline dorsal bite), 4 (lateral bite), and 5 (dorsolateral bite), and slightly increases in case 3 (anterior bite) for the pleurodire *E. madagascariensis* and case 2 (posterior bite) for *Proganochelys*. A large reduction is observed only in the cases in which the load is applied around the region of the attached element, i.e. the epiplastral processes anteriorly for *Proganochelys* (case 3) and the pelvic girdle posteriorly for *Proterochersis* and *E. madagascariensis* (case 2). In both these cases, the stress magnitude distributions show a shift towards higher stress when the shell connection is removed (Fig. 2b). The mean and distribution of von Mises stress (VMS) are strikingly similar between *Proterochersis* and *E. madagascariensis*, despite their different shell shapes (Fig. 1a). The mean stress pattern is very similar between the cryptodire and those of *Proterochersis* and *E. madagascariensis* (Fig. 2a). Interestingly, the stress magnitude distributions of the cryptodire *T. callirostris* are more similar to those of the detached *Proterochersis* and *E. madagascariensis* than to their attached models’ profiles (Fig. 2b). *Proganochelys* models stand out as considerably more stressed than all the other models, except in case 4 (lateral bite) in which its stress magnitude approaches that of the others.

**Fig. 3:**
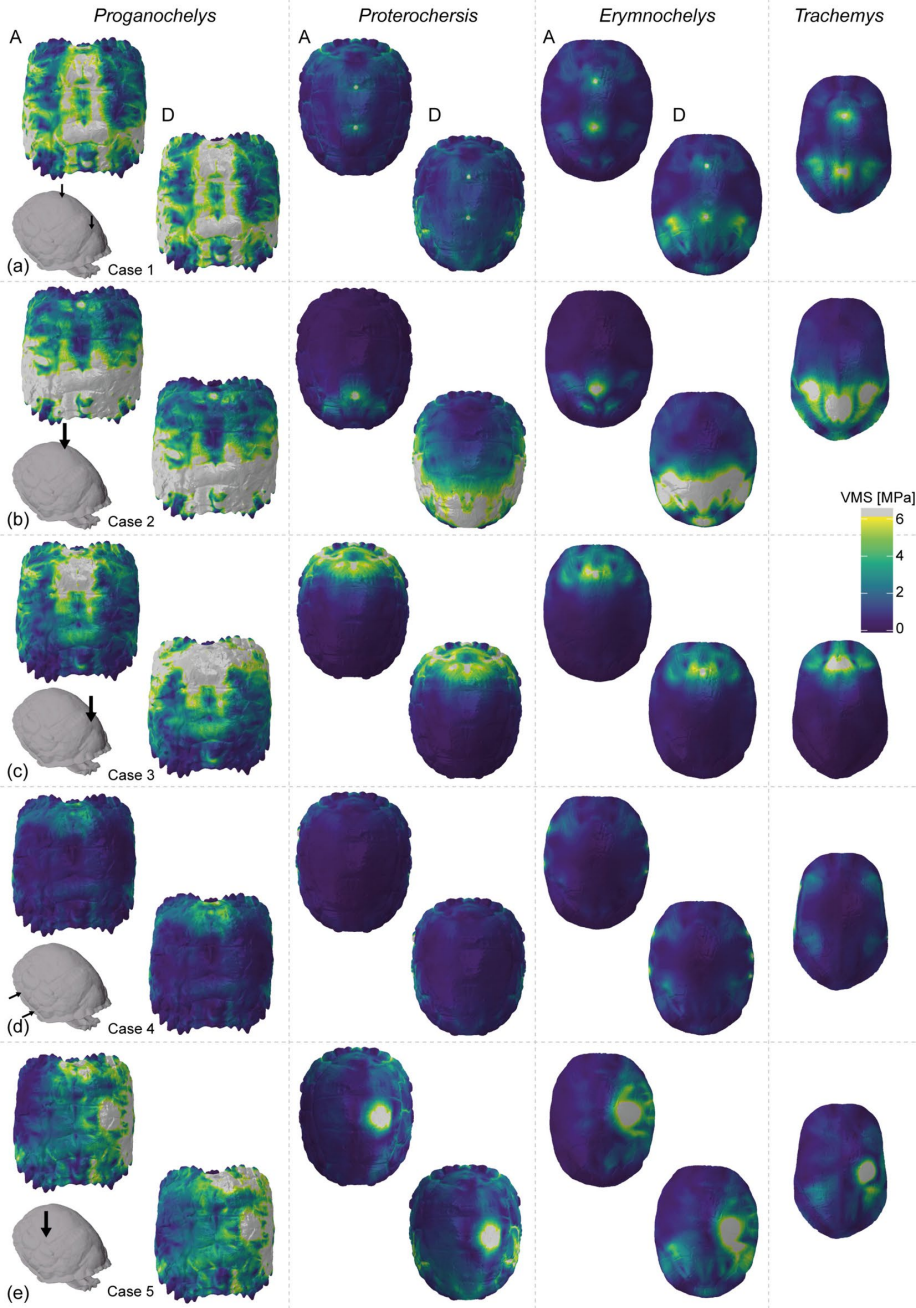
von Mises stress (VMS) contour plots in dorsal view of the studied taxa, subjected to different predation scenarios (a-e, cases 1 to 5). Colourmaps are scaled to 6 MPa peak VMS, with values above that represented in grey. For each case, the left top and bottom right plots of each taxon show the results of attached (A) and detached (D) models.

**Fig. 4:**
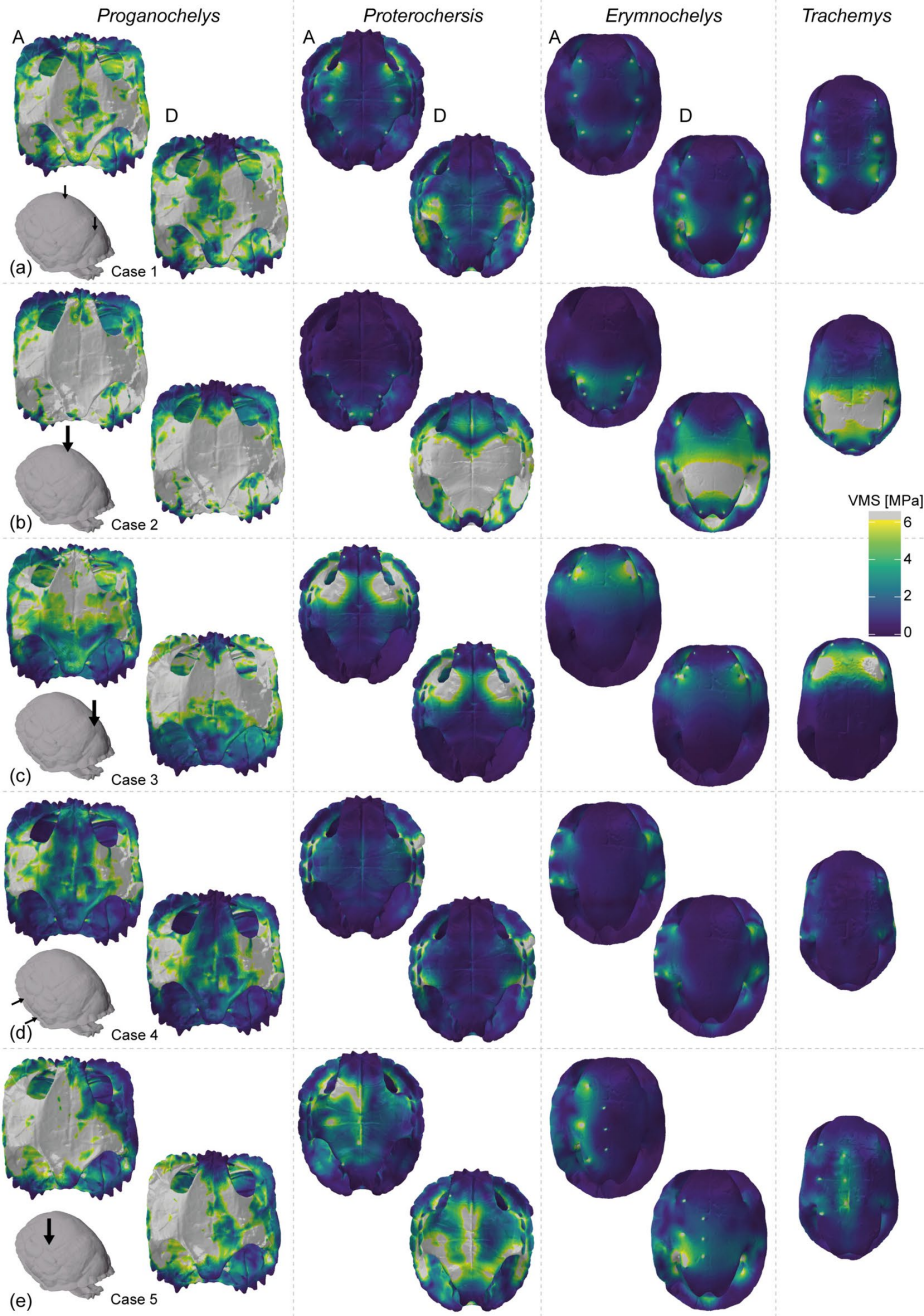
von Mises stress (VMS) contour plots in ventral view of the studied taxa, subjected to different predation scenarios (a-e, cases 1 to 5). Colourmaps are scaled to 6 MPa peak VMS, with values above that represented by grey shades. For each case, the left top and bottom right plots of each taxon show the results of attached (A) and detached (D) models.

The VMS contour plots show very similar stress distribution in all taxa (Figs. 3, 4). As a general pattern in all tested taxa and cases, highly stressed areas occur around the loaded and constrained nodes and on the shell bridge. In all cases, the detached model of *Proganochelys* shows a stress reduction in the anterior tip of the plastron (Fig. 4a–e), but an overall increase in the carapace (Fig. 3a–e). For *Proterochersis* and *E. madagascariensis*, differences in VMS distribution between the attached and detached models are apparent only in midline dorsal (case 1), anterior (case 2), and laterodorsal bites (case 5), in which the region around the inguinal buttresses is less stressed in the attached in comparison to the detached models (Fig. 4a, b, e). This reduction is also accompanied by increased stress around the axillary buttresses in cases 1 and 5 for *Proterochersis* and only in case 5 for *E. madagascariensis*. Interestingly, the inguinal buttresses and posterior half of the plastron and carapace are more stressed than the anterior parts in *T. callirostris* in cases 1 and 5 (Fig. 4a, e), making it more akin to the detached models of *Proterochersis* and *E. madagascariensis* in those cases. The largest difference between attached and detached models of *Proterochersis* and *E. madagascariensis* is observed in the posterior bite (case 2; Figs. 3b, 4b), in which the entire posterior half of the shell is highly stressed in the detached models, whereas high stress is limited to the areas adjacent to the constrained and loaded nodes in the attached models. In case 2, the stress distribution in the cryptodire *T. callirostris* is somewhat intermediate between the attached and detached models of the pleurodire *E. madagascariensis* and *Proterochersis*.

### Ecomorphological analysis

**Fig. 5:**
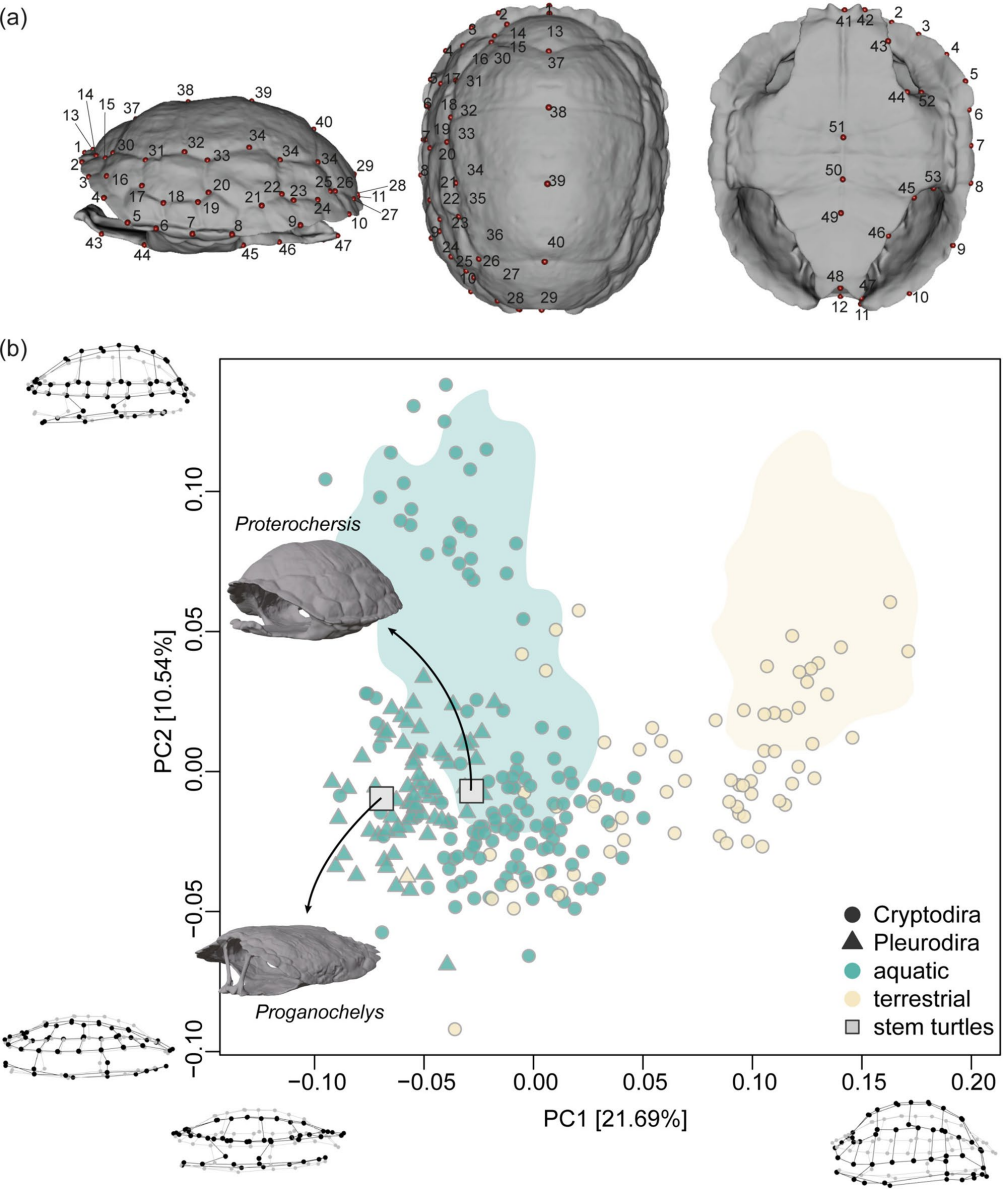
(**a**) Landmarks used to quantify shape variation in turtle shells (based on^48^ plotted on the 3D model of *Proterochersis*. (**b**) Morphospace plot based on the first two PCs representing shell shape variation in turtles. The positions of the models of *Proganochelys quenstedtii* and *Proterochersis* sp. are highlighted by squares. Landmark configurations representing shape extremes (black dots and lines) related to variation along the PC1 and PC2 axes superposed to the mean shape (grey dots and lines) are shown on the corners of each axis. Green and beige shaded areas represent the optima associated with aquatic and terrestrial taxa, respectively^43^.

We included our models of the shells of *Proterochersis* sp. and *Proganochelys quenstedtii* in a previously published 3D geometric morphometrics dataset^43,44,48^. This dataset was used to investigate how well three functional variables—self-righting ability, hydrodynamics, and strength—can predict shell shape in extant turtles^43^. By including the two early stem-turtles we aimed to provide some additional insights on their palaeobiology based on their position in the phenotypic morphospace. In comparison to the original analyses (which were shown to contain ecomorphologically relevant signals only in PC1 and PC2^43^), the variance in the principal components (PCs) changed only slightly. Whereas in the previous analyses, PC1 and PC2 explained respectively 21.9% and 10.6% of the total variation, in our results they relate to 21.7% and 10.5% of the variation, respectively (Fig. 5b). The shape changes related to each of the first two PCs are also very similar (Fig. 5b): shells in the bottom left quadrant (PC1-, PC2-) represent flatter phenotypes whereas the upper right quadrant (PC1+, PC2+) is populated by high-domed, rounder shells with broader plastra. There is virtually no change in the distribution of the extant species on the resulting morphospace in relation to the previous results^43,44,48^. Terrestrial and aquatic turtles show different distributions, although with considerable overlap in the centre of the morphospace. Terrestrial turtles spread diagonally on the morphospace from slightly negative to positive values of the first two PCs and aquatic taxa are distributed vertically along negative to positive PC2 values and are mostly restricted to lower values of PC1 (Fig. 5). Both *Proganochelys quenstedtii* and *Proterochersis* plot on slightly negative values of PC1 and PC2, thus largely contained within the aquatic area of the morphospace (but within the overlapping area). Linear discriminant analyses considering two categories (aquatic vs. terrestrial) show a strong aquatic signal for both stem-turtles (99.80% and 98.78% for *Prog. quenstedtii* and *Proterochersis*, respectively) when the first five PCs are analysed (= 53.7% of the total variation, correct classification rate: aquatic = 99.51%; terrestrial = 71.01%)—the phenotypic variation related to each PC can be seen in Stayton et al.^48^—, but they tend to have stronger terrestrial signals (nearly 100%) when higher percentages of the variation are considered (about 95% of the variation, i.e. PCs 1 to 59, correct classification rate: aquatic = 99.51%; terrestrial = 92.75%). Using three classes (predominantly aquatic, semiaquatic, and terrestrial) does not change much the results (see electronic supplementary file), although the correct classification rate decreases for the non-terrestrial classes. With PCs 1 to 5 (correct classification rate: predominantly aquatic = 67.57%; semiaquatic = 89.31%; terrestrial = 71.01%) *Prog. quenstedtii* is predicted as a predominantly aquatic turtle (90.41%) and *Proterochersis* as semiaquatic (75.73%). Analysing PCs 1 to 59 (correct classification rate: predominantly aquatic = 89.19%; semiaquatic = 96.95%; terrestrial = 92.75%) again predicts a terrestrial classification (nearly 100%) for both stem-turtles.

## Discussion

Comparisons of our attached and detached models of *Proganochelys quenstedtii* show that suturing the epiplastral processes does not affect its mean stress or stress distribution in this taxon (Figs. 2, 3, 4). The mean stress values only decrease when the load is applied anteriorly around the attached elements (i.e., case 3). However, even in this case, the decrease in mean stress is relatively small and both the von Mises stress (VMS) contour plots and magnitude distributions are nearly indistinguishable between the attached and detached models (Figs. 2, 3, 4). Thus, we conclude that load-bearing performance was not the main selection factor behind the evolution of sutured epiplastral processes in early stem-turtles.

Epiplastral processes sensu stricto are autapomorphic to Testudinata (i.e., turtle-like shelled reptiles), but its homologies are still poorly understood. The long and slender clavicles of the proto-turtles (Fig. 1) *Eunotosaurus africanus* and presumably *Pappochelys rosinae* articulated with the interclavicle medially and the cleithra dorsally^23,24^, as is the case in many other early tetrapods^49,50^. whereas *Odontochelys semitestacea* already displays a fully ossified plastron, which incorporates the interclavicle and clavicles^21^. In most Triassic Testudinata, such as *Proganochelys quenstedtii* and the australochelyids *Palaeochersis talampayensis* and *Waluchelys cavitesta*, the clavicles (now the epiplastra) still maintain the plesiomorphic connections to the interclavicle (entoplastron) medioventrally and the cleithrum (incorporated into the carapace as the nuchal) dorsally, the latter achieved by the dorsal process of the epiplastron^13,16,17^. The only exception among early testudinates (Fig. 1) is proterochersids (*Proterochersis* spp., *Keuperotesta limendorsa*), in which although the epiplastral processes are present and can be relatively long^26^, they do not contact the carapace. The epiplastral processes are supposedly lost in Mesochelydia^11,13^, but short dorsal processes of the epiplastron are still found in some mesochelyids, e.g., *Kayentachelys aprix*^51^, *Heckerochelys romani*^52^, *Meiolania platyceps*^53^, and *Eileanchelys waldmani*^54^.

Given the lack of support for the load-bearing hypothesis (Figs. 2, 3, 4), we propose the epiplastral processes are a plesiomorphic retention of the ancestral rod-like dorsal process of the clavicles found in many early tetrapods and diapsids^16,49,55–57^. Epiplastral processes are found in early testudinates even without reaching the carapace^24,26,51,54^, and also in proto-turtles without a fully formed carapace (i.e. *Odontochelys semistestacea*^21^). The epiplastral processes in stem-turtles maintained the plesiomorphic connection between the modified clavicles and the cleithra dorsally (Fig. 1) and, most likely, also the topological relation to muscles related to the neck and head, such as the *musculus sternocleidomastoideus* (= *episternocleidomastoideus*^56,57^, *plastrocapitis*^58^ and many other names^2,49,58^. The *m. sternocleidomastoideus* originates in the back of the skull and inserts to the anterior face of the dorsal projection of the clavicle^56–59^. In extant turtles, this muscle has shifted its insertion ventrally, to the epiplastra and entoplastron^2,60^. Although it has been suggested that this ventral shift occurred early in the evolution of Testudinata^60^, we hypothesize that in taxa with large dorsal epiplastral processes (such as in *Proganochelys quenstedtii*) the ancestral insertion was maintained and that the ventral migration towards the dorsal surface of the plastron occurred together with the reduction of the dorsal processes. This hypothesis explains why the dorsal epiplastral processes are ancestral to testudinates (and *O. semitestacea*), as well as their gradual reduction and the lack of relation to shell strength.

Attached and detached pelvis models perform strikingly alike in pleurodires and *Proterochersis* sp. (Figs. 2, 3, 4). Attaching the pelvic girdle decreased mean stress in similar proportions in all tested cases for the *Proterochersis* and *Erymnochelys madagascariensis* models, and also for a model of *Pelomedusa subrufa* analysed previously^36^. The strongest difference between detached and attached models (Fig. 2a) occurs when the load is applied posteriorly around the area where the pelvic girdle is attached (i.e. case 2). Case 3 (anterior bite) shows an increase rather than a decrease in mean stress for *E. madagascariensis* from the detached to the attached models, but the difference is very small and likely not meaningful considering this is also the case in which the simulations of *Proterochersis* and *Pe. subrufa*^36^ yielded the smallest difference (Fig. 2a) between detached and attached models. The VMS distribution in the shell—assessing both the stress magnitude distribution and the contour plots—is nearly identical between *Proterochersis* and *E. madagascariensis* (Figs. 2b, 3, 4). These results imply a comparable performance of the attached pelvic girdle in pleurodires and *Proterochersis* sp. Therefore, not only the morphology^24^ but also an analogous function is expected to have evolved at least twice in Testudinata.

The pelvis of proterochersids attaches to the carapace via broadened and elongated dorsal surfaces of the ilia and to the plastron via the lateral pubic processes, a sagittal keel of the puboischiadic plate, and triangular bases of the ischia, with a minor contact of the epipubic process observed in some individuals^25,26^. As such, the contact area is more substantial and the posterior part of the carapace received more support than in crown- and stem-pleurodires^38,40,61^. The mechanical function of the pelvis as a support for the posterior part of the carapace appears at first a plausible hypothesis, especially because in *Proterochersis* spp.—unlike in later testudinatans which have small number of costals, suprapygals, and pygal— this region was revealed to be composed of a mosaic of small, irregular ossifications, which might have potentially acted as a weak point, especially prior to shell ankylosis^11,19^.

Our results, nevertheless, do not support shell strengthening as the main function of the attached pelvis. Although a small decrease in mean stress is observable from the detached to the attached models of *Proterochersis* and the pleurodires in all tested cases (Fig. 2a), the stress magnitude distribution and the VMS contour plots (Figs. 2, 3, 4) are virtually identical in both scenarios. Only in case 2 (posterior bite) a noteworthy change is observed in both mean stress and VMS distributions, but this is expected given the resistance to axial compressive forces and load redistribution behaviours of columnar structures^62^. Moreover, it can be argued that even the reduction in mean stress is low in all cases when the load is not applied near the pelvic girdle. If the main function of an attached pelvis was to increase shell strength against predator attacks—as argued particularly for flatter pleurodires^36^—one would expect a general effect, and not only a local improvement posteriorly. Predatory biases towards posterior attacks could explain the reinforcement of this shell region. However, targeted attacks usually focus on soft parts and not particular shell regions^63^ and, even if that was the case, associated selective pressures should be expected to affect equally all aquatic turtles inhabiting similar environments^64^, as in the case of convergent evolution in both pleurodires and cryptodires of a hinged plastron associated with protecting the head and neck^9,65^. In other words, to convincingly support the increased load-bearing hypothesis an effect not only in mean stress values but also in VMS distribution would be predicted in all tested cases. This does not mean that there is no improvement in terms of shell strengthening with the addition of an attached pelvis. This might indeed have allowed pleurodires to evolve their (on average) flatter shell phenotypes^37^. However, we argue that this relationship is a consequence of the plesiomorphically sutured pelvis in pleurodires. In our view, the trigger for attaching the pelvis to the shell was not related to increasing structural strength.

The ecology of Triassic stem-turtles remains subject to debate. The non-shelled taxa are generally considered aquatic^12,21,22,66–71^, but a terrestrial ecology is also deemed possible for *Pappochelys rosinae* and *Odontochelys semistestacea*^12,72^. Conflicting interpretations were published for the Triassic Testudinata. *Proterochersis* spp. were considered terrestrial, based on their high-domed shell^15^ and bone histology^73^, but shell geometry^66,74^, histology^19^, limb anatomy^26^, and potential bromalites^75^, suggests an aquatic ecology, at least for *Proterochersis porebensis*^19,26^. *Proganochelys quenstedtii* on the other hand, despite suggestions of aquatic ecology^16,29,66,74^, is more often interpreted as a terrestrial stem-turtle^28,69,73,76–79^. And, finally, limb anatomy suggests either a terrestrial^27,28,69,77^ or aquatic^66^ habitat for *Palaeochersis talampayensis*. This ambiguity likely stems from the fact that many correlates used to determine the ecology of crown-turtles (e.g. shell and limb bone structure and shape) might have not yet been fully established during the Triassic, still evolving through the transition to a shelled body plan. Moreover, Triassic testudinatans already exhibited diverse morphologies, growth strategies, and presumably ecologies^26,28,80^, challenging more straightforward palaeobiological inferences.

Recent approaches using macroevolutionary adaptive landscapes (also known as performance surface analysis^42–44^) have shown that the shell morphology of extant turtles can be successfully predicted by some functional traits—e.g. strength, hydrodynamics, and self-righting ability. Because the geometries of the shells of *Proganochelys* and *Proterochersis* have thus far yielded ambiguous signals regarding their palaeoecology^66,74,81,82^, here we took a first step to understand the ecomorphology of extinct testudinatans using performance surfaces by inserting the shell models of those two taxa in an existing database^43,44^. We acknowledge that the ideal approach would be to recalculate the performance surfaces based on the new morphospace. However, since the change in sample size (from N = 2724 to 2726) and the resulting variation explained by the first two PCs (used to obtain the performance surfaces) were minimal (0.21 in PC1 and 0.06 in PC2), we consider our approach valid at least for an approximation of their performance. Future studies including more extinct taxa should consider recalculating the performance surfaces.

*Proterochersis* plots in an area characterized by stronger shells than *Proganochelys* (Fig. 3b), matching our FEA results that show a weaker shell in the latter in all cases (Figs. 1, 2). Both stem-turtles occupy the region populated by most aquatic turtles and near their combined surfaces optimum (Fig. 3)^43,44^. This area is represented by flatter phenotypes and is more associated with the hydrodynamic performance peak (towards PC1- and PC2-) than with that of self-righting ability (towards PC1+ and PC2+). Terrestrial turtles conversely are distributed along a diagonal axis (negative-to-positive PC1 and PC2; Fig. 3b). This has been interpreted as the result of evolving free of the selective pressure for hydrodynamics^43^, whereas the phenotype of aquatic turtles seems more constrained by the performance of this functional trait. Although the performance surfaces suggest an aquatic signal for *Proganochelys*, we consider the evidence from other sources (such as limb morphology^69,77^, shell bone histology^73^, neuroanatomy^78,79^, and depositional context^72^) strong enough to contradict this result. Moreover, some aquatic and terrestrial species that present cryptic behaviours—such as hiding under leaf-litter (*Platemys platycephala*) or rocks (*Malacochersus tornieri* and *Platysternon megacephalum*)^83^—plot in this flatter-phenotype region of the morphospace (and are also wrongly predicted as aquatic taxa using LDA with the first five PCs). This could also be the case for *Proganochelys quenstedtii*^84^, however its much larger size (shell up to about 60 cm^16,85^) does not favour this hypothesis. Its spiky marginals, gular and extragular projections, and neck, limb, and tail osteoderms (the latter forming a club^16,85^) imply that deterring predator attacks was the main defensive strategy of *Prog. quenstedtii*. Its flat and large body with a low centre of gravity, far peripherally located potential pivot points, and prickly borders could preclude easy rolling and turning over, which might have been already efficient protection against terrestrial predators in Europe’s Triassic.

Yet, we consider the aquatic hypothesis much stronger for *Proterochersis*. The results of the morphospace analysis (Fig. 3) and associated performance surface^43,44^ are backed by an increasing amount of supporting evidence for an aquatic palaeoecology^19,26,66,74,75,82^. Here we submit that the attached pelvic girdle also constitutes strong support for this hypothesis. Attaching the pelvis to the shell limits femoral protraction, reducing the amplitude of movements that are important for locomotion on land^33,34^, but it has been shown that the stiffened pelvic girdle stabilizes the hindlimb in pleurodires improving their swimming performance in comparison to cryptodires^35^. Thus, we hypothesize that selective pressure for improved swimming performances associated with the aquatic habitat was the trigger for attaching the pelvic girdle in both the pleurodiran and *Proterochersis* lineages, consisting in strong evidence for the interpretation of this early taxon as an aquatic stem-turtle.

## Material and methods

### Specimens and data acquisition

We analysed two representatives of early (Late Triassic) stem-turtles, *Proganochelys quenstedtii* and *Proterochersis* sp. (Proterochersidae), and compared them to an extant cryptodire, *Trachemys callirostris*, and a pleurodire, *Erymnochelys madagascariensis*. The digital models of *Proganochelys quenstedtii* (SMNS 16980), *Trachemys callirostris* (SMF 7498), and *Erymnochelys madagascariensis* (SMF 33056) were based on single specimens. Given that there is no completely prepared undistorted specimen of *Proterochersis* spp., we created a composite digital model of this taxon based on specimens of *Prot. robusta* (SMNS 17561, SMNS 56606) and *Prot. porebensis* (ZPAL V. 39/48, ZPAL V. 39/49). Using specimens of both species was necessary, given the incomplete information on the pelvis and the visceral surface of the carapace and plastron of *Prot. robusta*, and the absence of non-deformed specimens of *Prot. porebensis.* We additionally used the dorsal vertebrae of *Prog. quenstedtii* (SMNS 16980) to complete the model as these vertebrae are not well preserved in any *Proterochersis* spp. specimen. We used SMNS 17561 as a scaffold for retrodeforming the other models containing visceral information.

We employed either photogrammetry or structured-light surface scanning to digitise the specimens, based on their complexity and size, and the local availability of the tools. The shells of *Proganochelys quenstedtii* and *Proterochersis* spp*.* were all digitised using photogrammetry with a Nikon D5100 (ZPAL V. 39/48, ZPAL V. 39/49) or Canon EOS M6 mark II (SMNS 16980, SMNS 17561) cameras. To assemble the models from the photographs we used Metashape Pro (v. 1.8.4.14856, Agisoft), and the surface models were exported as .stl files. The broken-off dorsal epiplastral processes of *Prog. quenstedtii* SMNS 16980, as well as *Prot. robusta* SMNS 56606 were digitized using the Shining 3D EinScan Pro 2X surface scanner with a tripod and Ein-Turntable (alignment based on features), and EXScan Pro 3.2.0.2 software. The specimens of *T. callirostris* and *E. madagascariensis* were digitised using an Artec Spider handheld scanner with the software ArtecStudio 14 Professional (Artec 3D).

For the reconstruction of *Proterochersis* spp. we used the open-source 3D modeling software Blender (v. 3.4.1). Using the undeformed specimen SMNS 17561 as a reference, we retrodeformed the other carapace and plastron models. We downscaled SMNS 17561 to fit the proportions of specimen ZPAL V. 39/48 following its posterior and left lateral non-deformed sides. We processed the carapace, the plastron, and the pelvis separately. All specimens used were split along the midline and only the best-preserved side was manipulated and then duplicated, mirrored and merged with the other half. After isolating the left inner structure of the deformed specimen ZPAL V. 39/48 we merged it with the right outer surface of the reference specimen to form the carapace. Because no undeformed thoracic vertebral column is known for *Proterochersis* spp., we smoothed out the vertebrae present in ZPAL V. 39/48 and replaced it with the undeformed column of *Prog. quenstedii* (SMNS 16980), which we manipulated in scale and curvature to fit the original placement. This was a pragmatic solution justified by the lack of data and morphological similarity between the thoracic vertebrae and ribs of those taxa^25^. For the plastron restoration, the left part of specimen ZPAL V. 39/49 was aligned and retrodeformed to fit the curvature of the reference. Two original specimens were chosen for the reconstruction of the pelvis. The right ischium and ilium of SMNS 56606 and the left part of the pubis of ZPAL V. 39/49 were isolated and aligned at the posterior region of the reconstructed plastron and then merged. The epipubic process was sculpted based on previous reconstructions^11^ and openings, such as the obturator foramen and sacrovertebral canals, were sculpted open. Because in *Proterochersis* spp. the dorsal processes of epiplastra do not reach the carapace and their exact height and angle are unknown, we did not model them. Finally, cracks and holes were filled out to complete the restoration adding faces using Blender’s “Edit Mode”. The three parts were aligned based on the reference specimen and merged into one object. We then exported the reconstructed model as an STL file to retopologise and remesh it into an isotropic triangular mesh using the open-source software InstantMeshes^86^.

### Functional simulations

The surface models for each taxon were remeshed to reach about 600,000 triangular elements in Blender. To make the different simulations comparable across all models, we scaled all of them to the same straight carapace length (SCL = 200 mm), given that SCL is a common proxy for body size in testudinatans^87,88^ and this value is similar to the size of the specimens analysed in Williams and Stayton^36^. Because the shell of *Proterochersis robusta* is considerably higher and more massive than the other models, we further downscaled it to 95% of its original size (reaching SCL = 190 mm) to match the area of the other models. We remeshed the surface models in Hypermesh 13.0 (Altair Engineering) using the *tetramesh* function, with the options “Optimize Mesh Quality” and “Gradual” to obtain a solid mesh and built the finite element (FE) models on the same software.

We followed a similar approach to that of Williams and Stayton^36^ to test the performance of the attached pelvic girdle in *Proterochersis* sp. and pleurodires. We conducted two sets of finite element analyses (FEA) for the *Proterochersis* and the *E. madagascariensis* models: the “attached” set using the original models in which the pelvis connects the carapace and the plastron; and the “detached” set, in which we deleted elements in the pelvic girdle, thus removing the connection between the dorsal and ventral portions of the shell. For each set, we applied a total load of 500 N on different positions to simulate five distinct predation cases, based on Williams & Stayton^36^, which used bite forces of common predators of turtles of about SCL = 200 mm. In case 1, ventrally oriented loads of 250 N were applied to two nodes on the carapace along the midline, one in the anterior half and one in the posterior half; in case 2, a single ventrally directed 500 N load was applied dorsally to the posterior half of the carapace; in case 3, a single ventrally oriented 500 N load was applied dorsally to the anterior half of the carapace; in case 4, we simulated a lateral bite, by applying two loads of 250 N directed medially on the lateral surfaces of the peripherals along the bridge region; and, finally, in case 5, one ventrally oriented load of 500 N was applied laterodorsally to the carapace halfway along the anteroposterior axis. Six nodes were constrained in all cases, always on the opposite side of the shell to which the loads were applied. Likewise, the effects of the dorsal epiplastral processes on shell performance were evaluated with attached and detached models of *Proganochelys quenstedtii*. For further comparison, we also simulated the same five cases in the cryptodire, which has no additional elements attached to the shell aside from the lateral bridge, totalling 35 FE models.

The FE models were solved using Abaqus 6.14 (Simulia). The results (Figs. 1, 2) were evaluated using mean stress per element calculated using 99% of the values^46,47^, stress magnitude distribution using ridgeline plots^45^, and contour plots of von Mises stress (VMS).

### Geometric morphometric analyses

To assess how well three functional variables—self-righting ability, hydrodynamics, and strength—explain the shell shapes of *Proganochelys* and *Proterochersis*, we used 3D geometric morphometrics to summarize their form into a morphospace comparable to those of Stayton^43,44^. We placed 53 landmarks (Fig. 3a), 40 in the carapace and 13 on the plastron, using Checkpoint (Stratovan Corporation) following the landmark concept of Stayton et al.^48^, used by Stayton^43,44^. Because the number of dermal scutes in early testudinatans differs from the modal scute number of crown-turtles, we displaced some landmarks to an adjacent position (Fig. 3a). It is important to note that the same approach was conducted in the original study because not all Testudines share the same number of scutes^48^. All analyses were conducted in R v. 4.3.0^89^. We ran a Generalized Procrustes Analysis^90^ on the 3D coordinates dataset using the *gpagen* function in the R package *geomorph* v. 4.0.5^91,92^, followed by a Principal Component Analysis (PCA) using the *procSym* function in the *Morpho* v. 2.11 package^93^. All plots were made using base R functions and colour-blind-friendly colours. A Linear Discriminant Analysis was conducted using the function *lda* in the *MASS* v. 7.3–60 package^94^ with a priori defined ecological categories (aquatic and terrestrial; see supplementary data) and three subsets of the PC scores: 100% (full dataset), 95% (first 59 PCs), and 50% (first five PCs) of the shape variation. A second version of the ecological categorization was also tested, using predominantly aquatic, semiaquatic, and terrestrial classes.

## Supplementary Information

Supplementary Information.

## Data Availability

All data including surface models, FEA results, and the R script for all analyses and plots can be found at the Figshare repository at 10.6084/m9.figshare.25650960.
