# Supplementary material for: Shell biomechanics suggests an aquatic palaeoecology at the dawn of turtle evolution
Source: Sci Rep. 2024 Sep 18;14:21822. doi: 10.1038/s41598-024-72540-7 (PMC11411134; doi:10.1038/s41598-024-72540-7)
Supplement: Supplementary file 1 — Supplementary Information. [file 41598_2024_72540_MOESM1_ESM.pdf]

Shell biomechanics suggests an aquatic palaeoecology at the dawn of turtle evolution

Eletronic Supplementary Material

Ferreira GS, Hermanson G, Kyriakouli C, Dróżdż D, Szczygielski T

Linear Discriminant Analysis (LDA)

Aquatic vs. Terrestrial classification

We performed LDA on about 50% of the total variation in the PC scores (i.e., PC1 to PC5) of the shell shape data and using a **habitat** variable containing two classes: **aquatic** and **terrestrial** .

```
lda.res = lda(PCA_mean_subset, grouping = species.data$Habitat)

lda.res.PC50 = lda(PCA_mean_subset[, 1:5], grouping = species.data$Habitat)

pred.PC50 = predict(lda.res.PC50)
```

This returned a correct classification rate of 0.995122 for the **aquatic** class and of 0.7101449 for the **terrestrial** class.

Using these results we then predicted the habitats of *Proganochelys quenstedtii* and *Proterochersis* based on their PC scores, using:

```
predict(lda.res.PC50, newdata = PCA_mean[c("Proganochelys",
                                           "Proterochersis"), 1:5])
```

```
$class
[1] A A
Levels: A T

$posterior
```

```
                         A          T
Proganochelys  0.9980258 0.00197419
Proterochersis 0.9878468 0.01215318

$x
                        LD1
Proganochelys  -0.9574702
Proterochersis -0.3507792
```

Both stem-turtles are predicted as aquatic under this model. We additionally used the same results to predict the habitats of three terrestrial but very flattened turtles: *Malacochersus tornieri*, *Platemys platycephala*, and *Platysternon megacephalum*, which also turned out predicted as aquatic:

```r
predict(lda.res.PC50, newdata = PCA_mean[c("Malacochersus_tornieri",
                                           "Platemys_platycephala",
                                           "Platysternon_megacephalum"), 1:5])
```

```
$class
[1] A A A
Levels: A T

$posterior
                                  A            T
Malacochersus_tornieri    0.9922267 0.0077732958
Platemys_platycephala     0.9954961 0.0045038958
Platysternon_megacephalum 0.9999386 0.0000613673

$x
                                LD1
Malacochersus_tornieri    -0.5005929
Platemys_platycephala     -0.6828445
Platysternon_megacephalum -2.1103000
```

In a second analysis, considering 95% of the total variation (PCs 1 to 59) and the same habitat categorization,

```r
lda.res.PC95 = lda(PCA_mean_subset[, 1:59], grouping = species.data$Habitat)

pred.PC95 = predict(lda.res.PC95)
```

we obtained a correct classification rate of 0.995122 and 0.9275362 for the **aquatic** and **terrestrial** classes, respectively. And with this model, the predictions for the above-mentioned taxa are:

```
predict(lda.res.PC95,
        newdata = PCA_mean[c("Proganochelys",
                             "Proterochersis",
                             "Malacochersus_tornieri",
                             "Platemys_platycephala",
                             "Platysternon_megacephalum"), 1:59])
```

```
$class
[1] T T T A A
Levels: A T

$posterior
                                     A            T
Proganochelys             1.002656e-33 1.000000e+00
Proterochersis            1.688195e-27 1.000000e+00
Malacochersus_tornieri    9.933939e-07 9.999990e-01
Platemys_platycephala     9.999942e-01 5.815124e-06
Platysternon_megacephalum 9.994091e-01 5.908828e-04

$x
                                 LD1
Proganochelys             15.8118787
Proterochersis            13.1161335
Malacochersus_tornieri     4.1236206
Platemys_platycephala     -0.7421555
Platysternon_megacephalum  0.1268865
```

Proganochelys quenstedtii, *Proterochersis* and *Malacochersus tornieri* are predicted as terrestrial, whereas *Platemys platycephala* and *Platysternon megacephalum* are still predicted as aquatic.

Predominantly aquatic, semiaquatic, and terrestrial classification

The same set of LDAs were also performed using three classes of habitat: **predominantly aquatic**, **semiaquatic**, and **terrestrial**. Considering PC1 to PC5

```
lda.res.PC50.AST = lda(PCA_mean_subset[, 1:5],
                       grouping = species.data.AST$Habitat)

pred.PC50.AST = predict(lda.res.PC50.AST)
```

the rate of correct classifications for **predominantly aquatic** was 0.6756757 , for **semi-aquatic** was 0.8931298 , and, finally, for **terrestrial** habitat it was 0.7101449 . Similarly to the analysis with two classes, the predicted habitats for *Proganochelys quenstedtii* was **predominantly aquatic** and for *Proterochersis* **semiaquatic**. The other three taxa were also predicted as:

```
predict(lda.res.PC50.AST,
        newdata = PCA_mean[c("Proganochelys",
                             "Proterochersis",
                             "Malacochersus_tornieri",
                             "Platemys_platycephala",
                             "Platysternon_megacephalum"), 1:5])
```

```
$class
[1] A S A S A
Levels: A S T

$posterior
                                  A          S            T
Proganochelys             0.9041004 0.09552328 3.763262e-04
Proterochersis            0.2295678 0.75732208 1.311008e-02
Malacochersus_tornieri    0.5261550 0.46796186 5.883184e-03
Platemys_platycephala     0.3838606 0.61193749 4.201921e-03
Platysternon_megacephalum 0.9211949 0.07879517 9.960930e-06

$x
                                 LD1        LD2
Proganochelys             -1.4849092 1.62246181
Proterochersis            -0.3792071 0.02292557
Malacochersus_tornieri    -0.7275984 0.67920685
Platemys_platycephala     -0.7954260 0.24312308
Platysternon_megacephalum -2.5517790 1.07575944
```

Finally, using 95% of the variation contained in PCs 1 to 59,

```
lda.res.PC95.AST = lda(PCA_mean_subset[, 1:59],
                       grouping = species.data.AST$Habitat)

pred.PC95.AST = predict(lda.res.PC95.AST)
```

we obtained higher correct classification rates: 0.8918919 for **predominantly aquatic**, 0.9694656 for **semiaquatic**, and 0.9275362 for the **terrestrial** class, and the classifications for the five taxa were:

```
predict(lda.res.PC95.AST,
        newdata = PCA_mean[c("Proganochelys",
                             "Proterochersis",
                             "Malacochersus_tornieri",
                             "Platemys_platycephala",
                             "Platysternon_megacephalum"), 1:59])
```

```
$class
[1] T T T S S
Levels: A S T

$posterior
                                     A            S            T
Proganochelys             4.188219e-13 3.463154e-33 1.000000e+00
Proterochersis            1.090159e-16 3.710097e-27 1.000000e+00
Malacochersus_tornieri    3.786996e-12 9.472435e-07 9.999991e-01
Platemys_platycephala     1.639068e-05 9.999771e-01 6.538422e-06
Platysternon_megacephalum 4.511492e-06 9.993270e-01 6.685151e-04

$x
                                LD1        LD2
Proganochelys             7.0797816 20.4027849
Proterochersis            7.4904508 13.9746994
Malacochersus_tornieri    4.3624987  0.7330436
Platemys_platycephala     0.1171424 -1.7771361
Platysternon_megacephalum 1.0004248 -1.5568210
```

Hence, terrestrial for the both stem-taxa and *Malacochersus tornieri*, but still semiaquatic for *Platemys platycephala* and *Platysternon megacephalum*.

The morphospace with the species colored by the two-class habitat classifier (aquatic vs. terrestrial) is presented on Figure 5 of the main text. On Figure S1 below we present the same

PCA plot using the three habitats classification instead: predominantly aquatic, semiaquatic, and terrestrial.

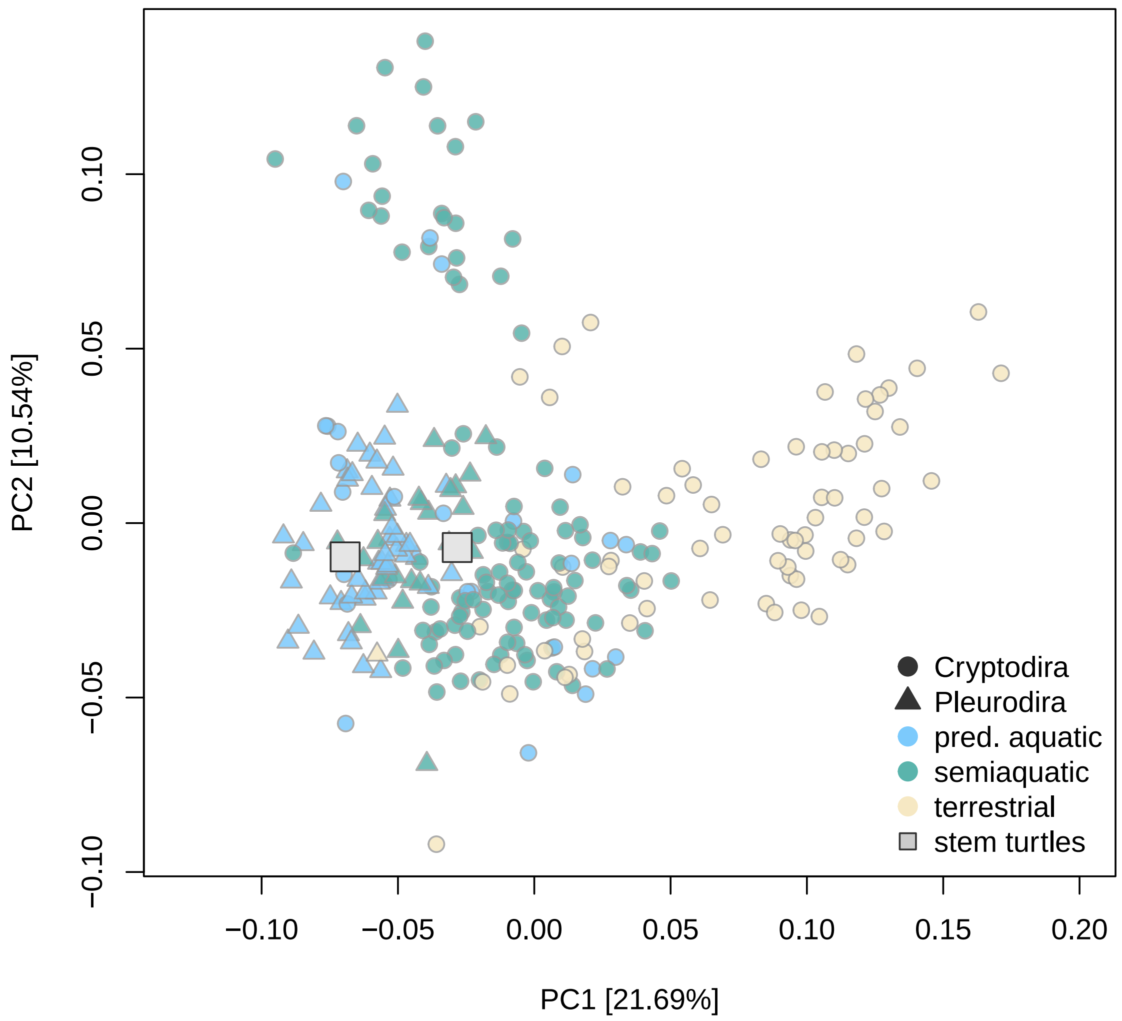

Figure S 1: Morphospace plot based on the first two PCs representing shell shape variation in turtles. The landmark configurations representing shape extremes can be found on Figure 5 of the main text.
